# Outcomes of Dual Mobility Bearings in Revision Total Hip Replacements

**DOI:** 10.7759/cureus.55585

**Published:** 2024-03-05

**Authors:** Christopher White, Waleed Abdalla, Prashant Awasthi, Farhad Iranpour, Padmanabhan Subramanian

**Affiliations:** 1 Department of Trauma and Orthopaedic Surgery, Imperial College Healthcare NHS Trust, London, GBR; 2 Department of Trauma and Orthopaedic Surgery, Royal Free London NHS Foundation Trust, London, GBR

**Keywords:** case series, dislocation, revision, total hip replacement, dual mobility bearing

## Abstract

Background

Dual mobility bearings have gained attention in the prevention of instability in revision total hip replacement. This study aimed to evaluate the use of dual mobility bearings in revision total hip replacement. The primary outcome was the rate of dislocation. Secondary outcomes included the rate of re-operation for any reason, surgical complications, serious medical adverse events, and 90-day mortality rate.

Methods

A single-centre case series of 55 consecutive operations in 49 patients who underwent revision total hip replacement using dual mobility bearings with a minimum follow-up of three months was studied.

Results

Early dislocation occurred in one case (2%), and there were no intra-prosthetic dislocations at a mean follow-up of 16 months. The rate of re-operation for any reason was 6/55 (11%) cases, and the post-operative infection rate was 2/55 (4%) cases. Serious medical adverse events occurred in 2/55 (4%) cases. The 90-day mortality rate was 1/55 (2%) cases. Two cases (2%) had cup abduction or anteversion angles outside of the safe zones although there were no dislocations in these patients.

Conclusion

This case series demonstrates a low dislocation rate in the early post-operative period for dual mobility bearings in revision total hip replacement. Dual mobility bearings show promise as an early low dislocation implant in revision total hip replacement. It remains to be determined whether dual mobility bearings are low-wear implants in the long term.

## Introduction

Total hip replacement is the most commonly performed adult reconstructive hip procedure, with nearly one million performed worldwide each year [1]. Increasing life expectancy has increased the number of procedures and consequently increased the requirement for revision total hip replacement surgery [2]. Over the last five years, the most common reasons for revision total hip replacement surgery were aseptic loosening, dislocation/subluxation, periprosthetic fracture, infection, and adverse soft tissue reaction to particulate debris [3].

Instability after a total hip replacement remains a significant cause of readmission and revision surgery [4,5]. The rate of instability after revision total hip replacement surgery has been reported to be as high as 9.8-25% [3,6-8]. The need for subsequent re-revision surgery is strongly associated with the time to the first revision, with 19.6% of total hip replacements revised within a year of primary surgery requiring re-revision within 10 years [3]. Techniques to treat instability in revision total hip replacement include modular component upsizing (increasing femoral head size), increasing femoral offset, use of constrained cups, and correcting component malposition [9].

**Figure 1:**
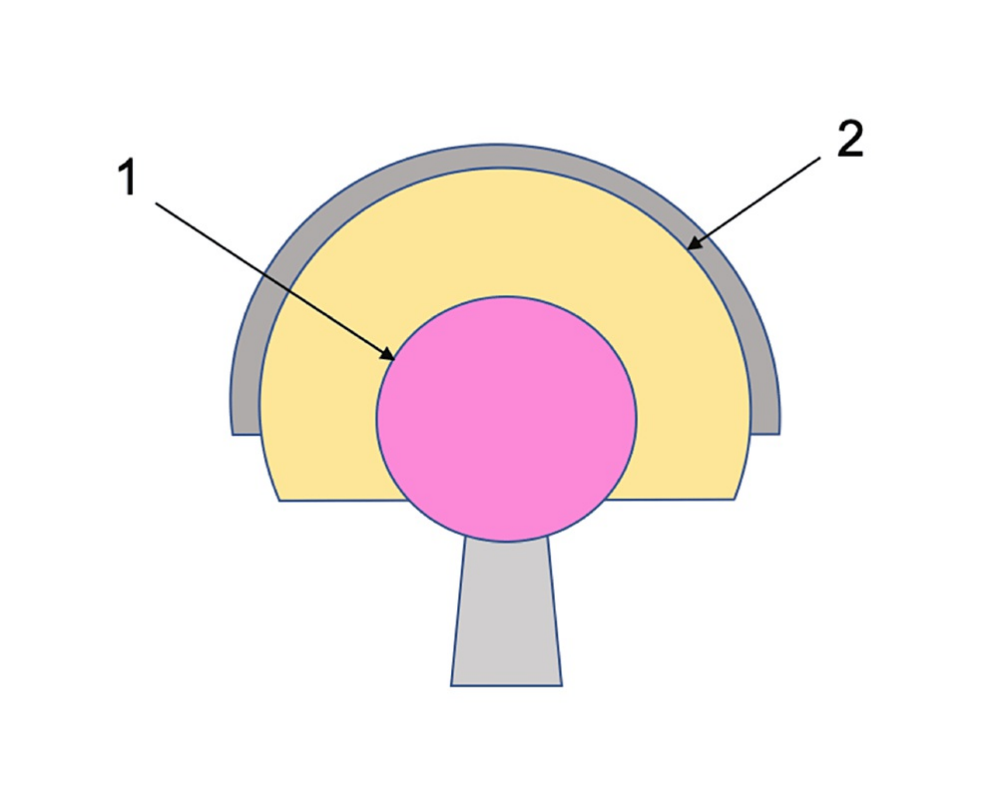
Diagram of a dual mobility bearing. 1: inner articulation between the femoral head and polyethylene liner; 2: outer articulation between the polyethylene liner and acetabular component

Dual mobility bearings have gained attention in the prevention of instability in revision total hip replacement. They consist of a smaller-diameter femoral head that articulates with a larger-diameter polyethylene liner, which in turn articulates with an acetabular component (Figure 1). This design increases the jump distance and impingement free range of movement. Reported outcomes of several studies using dual mobility bearings in revision total hip replacement support their effectiveness [10]. The rate of dislocation after revision surgery using dual mobility bearings has been reported to be as low as 0-3.5% [11]. Polyethylene wear was a significant problem for first-generation dual mobility bearings [12]. This problem appears to have lessened over the short and medium term through improved polyethylene fabrication, use of thinner and smoother trunnions, chamfered rims, and eccentric centres of insert and shell rotation [13-15]. Intra-prosthetic dislocation is an additional complication specific to dual mobility bearings and is characterised by dislocation of the outer polyethylene bearing surface from the inner femoral head, resulting in articulation between the inner femoral head and acetabular liner [16,17].

This study aimed to evaluate the use of dual mobility bearings in revision total hip replacement. The primary outcome was the rate of dislocation. Secondary outcome measures included the rate of re-operation for any reason, surgical complications, serious medical adverse events, and 90-day mortality rate.

## Materials and methods

A retrospective single-centre case series of 55 consecutive operations in 49 patients who underwent revision total hip replacement using dual mobility bearings was studied. Patients underwent surgery between 2019 and 2022. Inclusion criteria were patients undergoing revision total hip replacement with the use of dual mobility bearings with a minimum follow-up of three months.

Data was collected retrospectively using the Electronic Patient Record Systems and Centricity Picture Archiving and Communications Systems (PACS). Data collected included age, sex, body mass index, date of primary surgery and type of implant, any previous revision surgery, the indication for revision surgery and type of implant, peri-operative length of stay, blood transfusion requirement, requirement for critical care admission, and medical and surgical complications. A serious medical adverse event was defined as an untoward medical occurrence that resulted in prolonged hospitalisation or persistent or significant disability. Data was inputted and analysed using Microsoft Excel (Version 16.16.27).

Radiographic cup abduction and anteversion angles were measured using PACS on post-operative radiographs. The cup abduction angle was calculated as the angle formed by the intersection of a line passing through the inferior aspect of the radiographic teardrops and a line tangential to the rim of the cup on pelvic anteroposterior radiographs [18]. The cup anteversion angle was calculated as the angle between a line touching the opening surface of the acetabular component and a line perpendicularly drawn to the table on cross-table lateral hip radiographs (Woo and Morrey method) [19]. Acceptable ranges were defined as an inclination of 30-45 degrees and an anteversion of 5-25 degrees [20,21].

Patients undergoing elective surgery attended a pre-operative assessment clinic for pre-surgery optimisation. During surgery, patients were positioned in a lateral decubitus position, and a posterior approach to the hip was used. Patient preoperative skin preparation was performed using ChloraPrep (Becton, Dickinson). Repair of the capsule, short external rotators, and fascia was performed using a size 2 Vicryl suture (Ethicon). Skin closure was performed using size 3-0 Monocryl suture (Ethicon) (39/55), surgical skin staples (Medtronic) (15/55), and 3-0 Ethilon suture (Ethicon) (1/55). Revision procedures were performed by a fellowship-trained consultant orthopaedic surgeon specialising in revision hip surgery using a standard surgical technique. Patients received peri-operative antibiotic prophylaxis and post-operative venous thromboembolism prophylaxis.

The case series has been reported in line with the Preferred Reporting Of CasESeries in Surgery (PROCESS) Guideline [22].

## Results

The case series was composed of 23 men and 26 women with a mean age of 78 years (53-93 years) (Table 1). The mean body mass index was 27 kg/m^2^ (17-51 kg/m^2^). The mean follow-up duration was 16 months, and the median follow-up duration was 12 months (range: 3-36 months). There were no patients lost to follow-up. The mean time from the primary procedure to revision surgery was 10 years (one month to 32 years). Eleven patients underwent re-revision procedures and had already undergone a previous revision procedure. Indications for revision surgery were aseptic loosening (20/55) (36%), periprosthetic fracture (13/55) (24%), metallosis/adverse reaction to metal debris (9/55) (16%), infection (7/55) (13%), conversion of failed hemiarthroplasty (3/55) (5%), and dislocation (3/55) (5%). 

**Table 1:** Outcomes and demographic information for patients included in the case series. M: male; F: female; ARMD: adverse reaction to metal debris; TMAR: Trabecular Metal Acetabular Revision System; CPT: CPT Femoral System; Arcos ILS: Arcos interlocking stem; Arcos STS: Arcos splinted tapered stem

Age (years)	Sex	Body mass index (kg/m^2^)	Follow-up duration (months)	Indication for revision	Acetabular revision implant	Femoral revision implant	Post-operative dislocation	Re-operation	Surgical or medical complications	Red blood cell transfusion	Critical care admission	Length of stay (days)	90-day mortality	Cup abduction angle (degrees)	Cup anteversion angle (degrees)
89	F	23	14	Aseptic loosening	G7	CPT	-	-	-	-	-	6	-	31	11
86	F	23	9	Aseptic loosening	G7	CPT	-	-	-	-	Yes	7	-	35	13
86	F	28	12	Aseptic loosening	G7	CPT	-	-	-	-	-	19	-	36	30
86	F	34	35	Aseptic loosening	G7	Arcos ILS	-	Periprosthetic femoral fracture fixation	-	Yes	-	16	-	37	26
85	M	Not available	11	Aseptic loosening	G7	Arcos STS	-	-	-	-	Yes	34	-	40	15
85	M	24	10	Aseptic loosening	G7	Arcos ILS	-	-	-	-	Yes	13	-	30	25
84	F	Not available	24	Aseptic loosening	G7	Arcos ILS	-	Surgery for deep infection	-	-	-	39	-	30	25
84	F	27	19	Aseptic loosening	G7	CPT	-	-	-	-	-	7	-	35	25
84	M	27	26	Aseptic loosening	G7	Not applicable	-	-	-	Yes	-	7	-	35	25
81	F	Not available	15	Aseptic loosening	TMAR	Arcos STS	-	-	-	-	-	5	-	40	25
80	F	28	12	Aseptic loosening	Custom	Not applicable	-	-	-	Yes	Yes	14	-	40	25
77	F	Not available	11	Aseptic loosening	G7	Arcos STS	-	-	-	-	-	21	-	43	25
77	M	33	3	Aseptic loosening	G7	Not applicable	-	-	-	-	-	1	Yes	31	24
76	M	30	6	Aseptic loosening	G7	CPT	-	-	-	-	Yes	10	-	34	24
76	F	21	5	Aseptic loosening	G7	CPT	-	-	-	Yes	Yes	4	-	36	24
73	F	Not available	8	Aseptic loosening	G7	Arcos ILS	-	-	-	-	Yes	13	-	41	9
71	F	Not available	3	Aseptic loosening	G7	Arcos ILS	-	-	-	-	-	13	-	45	24
69	M	51	12	Aseptic loosening	G7	Arcos ILS	-	Staged revision for periprosthetic infection	Brachial artery thrombosis	Yes	-	14	-	31	23
67	F	30	13	Aseptic loosening	G7	CPT	-	-	-	-	-	5	-	31	23
65	F	29	26	Aseptic loosening	G7	Arcos ILS	-	-	-	-	-	7	-	32	23
89	F	23	7	Dislocation	G7	Arcos ILS	-	-	-	-	Yes	28	-	33	23
82	M	27	8	Dislocation	G7	Arcos ILS	-	-	-	-	Yes	4	-	35	23
61	M	31	15	Dislocation	G7	CPT	-	-	-	-	-	4	-	43	23
84	F	21	23	Failed hemiarthroplasty	G7	Not applicable	-	-	-	Yes	Yes	4	-	31	22
76	M	17	3	Failed hemiarthroplasty	G7	Arcos STS	-	-	-	-	-	2	-	33	22
72	F	19	10	Failed hemiarthroplasty	G7	Not applicable	-	Stem revision for periprosthetic femoral fracture	-	Yes	Yes	5	-	34	22
84	M	26	15	Infection	G7	Arcos STS	-	-	-	-	Yes	11	-	35	22
84	F	20	9	Infection	TMAR	CPT	-	Evacuation of haematoma	Deep vein thrombosis	Yes	-	58	-	38	22
82	F	20	36	Infection	TMAR	Arcos STS	-	-	-	-	-	21	-	44	22
79	F	22	11	Infection	G7	Total femoral replacement	-	-	Foot drop	Yes	Yes	50	-	33	21
72	F	28	33	Infection	G7	Proximal femoral replacement	-	-	-	Yes	-	21	-	35	21
71	M	37	7	Infection	G7	Arcos ILS	-	-	-	-	Yes	18	-	39	21
71	F	25	12	Infection	G7	Arcos STS	-	-	-	-	-	14	-	40	21
79	M	26	8	Metallosis/ARMD	G7	CPT	-	-	-	-	-	6	-	31	20
75	F	26	8	Metallosis/ARMD	G7	CPT	-	-	-	Yes	-	3	-	37	20
74	M	29	19	Metallosis/ARMD	G7	Arcos ILS	-	-	-	-	-	24	-	38	20
71	M	Not available	5	Metallosis/ARMD	G7	Not applicable	-	-	-	Yes	Yes	14	-	34	19
71	M	Not available	36	Metallosis/ARMD	G7	Arcos STS	-	-	-	Yes	-	13	-	36	12
70	F	28	7	Metallosis/ARMD	G7	Arcos STS	-	-	-	-	-	6	-	34	19
67	M	Not available	34	Metallosis/ARMD	G7	Not applicable	-	-	-	Yes	-	2	-	38	12
65	M	27	8	Metallosis/ARMD	G7	CPT	-	-	-	Yes	-	3	-	37	19
53	M	Not available	20	Metallosis/ARMD	G7	CPT	-	-	-	Yes	-	14	-	39	19
93	M	24	12	Periprosthetic fracture	G7	Not applicable	Yes	Closed reduction for dislocation	-	-	-	63	-	34	18
88	F	22	24	Periprosthetic fracture	G7	Arcos STS	-	-	-	Yes	Yes	33	-	42	15
88	F	32	9	Periprosthetic fracture	G7	Arcos STS	-	-	-	-	-	15	-	34	18
88	F	29	20	Periprosthetic fracture	TMAR	Not applicable	-	-	-	Yes	-	12	-	38	18
85	F	28	34	Periprosthetic fracture	G7	CPT	-	-	-	-	Yes	17	-	40	18
85	F	19	34	Periprosthetic fracture	TMAR	Arcos ILS	-	-	-	-	Yes	15	-	41	18
85	M	Not available	6	Periprosthetic fracture	G7	Arcos STS	-	-	-	-	-	25	-	41	18
83	M	30	10	Periprosthetic fracture	G7	Arcos STS	-	-	-	-	Yes	19	-	31	16
80	M	Not available	11	Periprosthetic fracture	G7	Arcos STS	-	-	-	-	-	14	-	36	16
77	F	26	13	Periprosthetic fracture	G7	CPT	-	-	-	-	Yes	10	-	32	15
76	F	27	26	Periprosthetic fracture	G7	Arcos STS	-	-	-		-	24	-	40	15
68	M	36	20	Periprosthetic fracture	G7	Arcos ILS	-	-	-		-	5	-	32	9
65	F	29	26	Periprosthetic fracture	G7	Arcos STS	-	-	-		-	8	-	42	13

Dual mobility bearings implanted were the G7 Dual Mobility (Zimmer Biomet) (49/55), Avantage Dual Mobility cemented into a Trabecular Metal Acetabular Revision System (Zimmer Biomet) shell (1/55) and cup-cage construct (4/55), and one custom acetabular dual mobility (Lima) (1/55) (Figure 1). Femoral head diameters were 28 mm (51/55) and 22 mm (4/55 in cup-cage constructs). Total hip replacement implants revised included hybrid, reverse hybrid, cemented, uncemented, and metal on metal including one hip resurfacing. Revision surgery included isolated acetabular component revision (9/55), femoral and acetabular component revision (44/55), proximal femoral replacement (1/55), and total femoral replacement (1/55). Femoral components were revised using the CPT Femoral System (Zimmer Biomet) (15/55), Arcos Modular Femoral Revision System (Zimmer Biomet) (29/55), and Megasystem-C Bone and Joint Revision System (Link Orthopaedics) (2/55).

One patient (2%) had a posterior dislocation at eight weeks post-operatively, which was treated successfully with closed reduction. This patient had undergone a recent revision procedure prior to re-revision to a dual mobility construct for instability. The patient had no further episodes of dislocation at 12 months follow-up. There were no intra-prosthetic dislocations.

Six patients (11%) underwent further surgery for the following reasons: evacuation of haematoma (1/55), closed reduction for dislocation (1/55), subsequent periprosthetic femoral fracture fixation (1/55), stem revision for periprosthetic femoral fracture (1/55), surgery for deep infection (1/55), and staged revision for periprosthetic infection (1/55). One patient (2%) sustained a foot drop after total femur replacement surgery. The post-operative infection rate was 2/55 (4%) cases. Serious medical adverse events occurred in 2/55 (4%) cases and were deep vein thrombosis (1/55) and brachial artery thrombosis (1/55). Twenty-one patients (38%) required a peri-operative red blood cell transfusion, and 20 patients (36%) were admitted to critical care post-operatively. The mean peri-operative length of hospital stay was 15 days (1-63 days). The 90-day mortality rate was 1/55 (2%) cases. 

**Figure 2:**
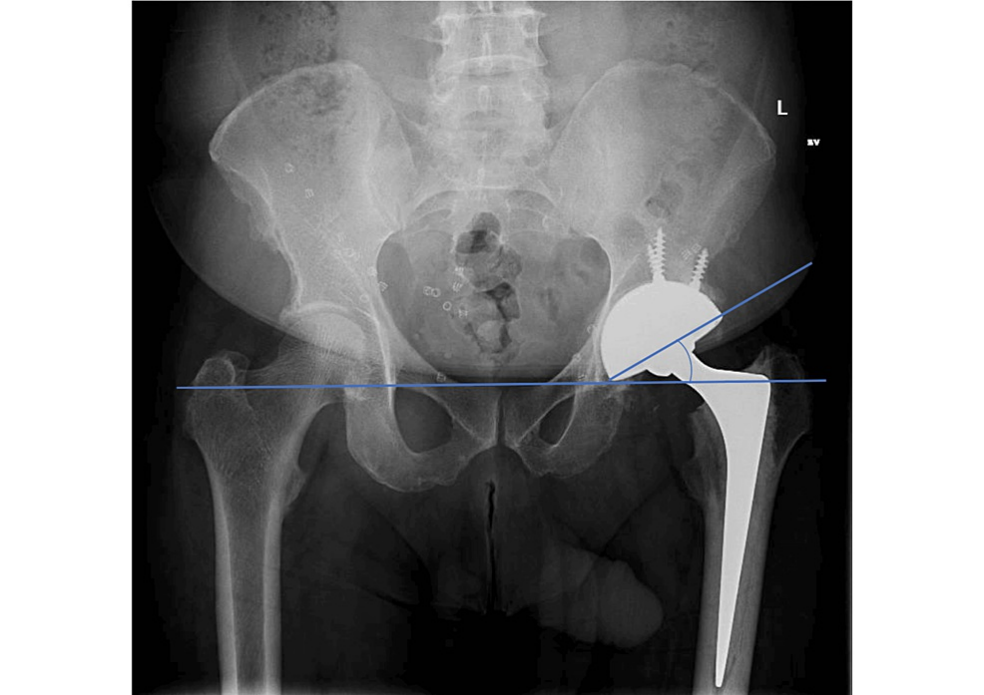
Radiographic cup abduction angle measurement. The cup abduction angle was calculated as the angle formed by the intersection of a line passing through the inferior aspect of the radiographic teardrops and a line tangential to the rim of the cup on pelvic anteroposterior radiographs.

**Figure 3:**
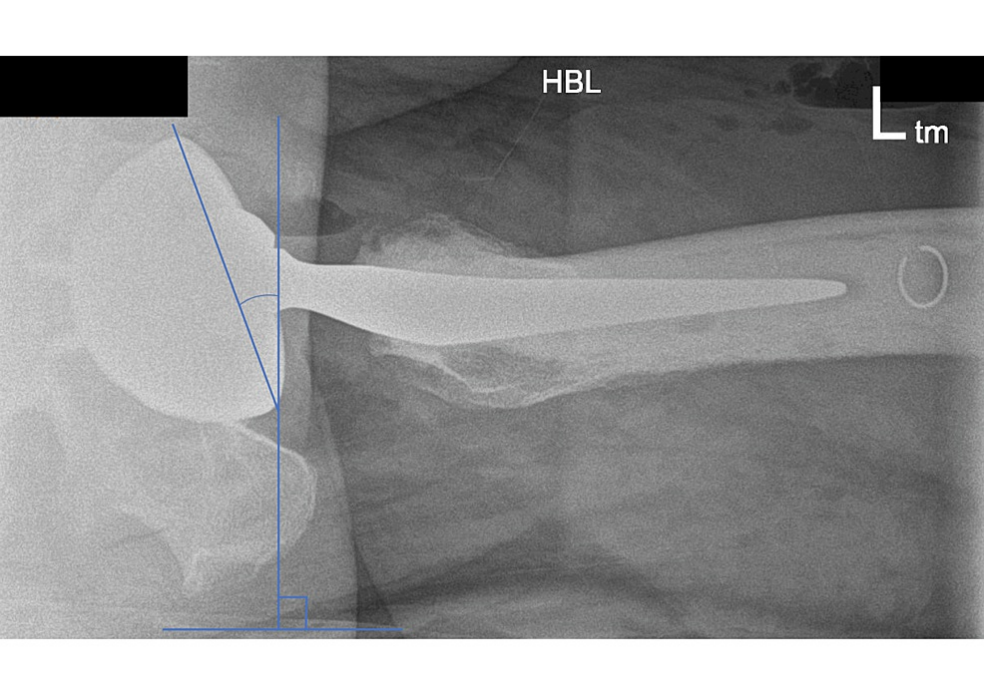
Radiographic cup anteversion angle measurement. The cup anteversion angle was calculated as the angle between a line touching the opening surface of the acetabular component and a line perpendicularly drawn to the table on cross-table lateral hip radiographs.

**Figure 4:**
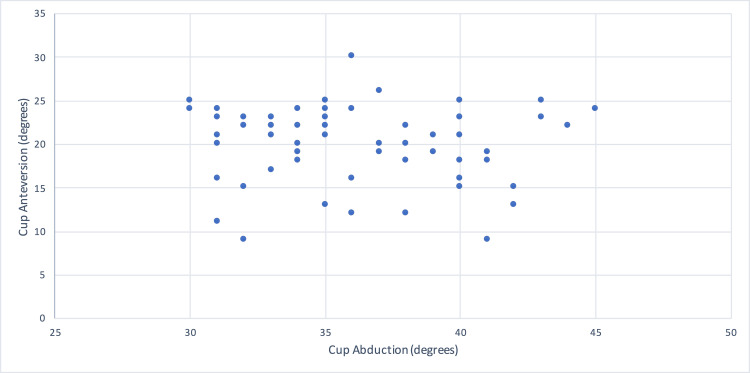
Scatterplot of radiographic cup abduction and anteversion angles for the 55 operations in this case series.

The mean radiographic cup abduction angle was 36 degrees (30-45 degrees), and the mean radiographic cup anteversion angle was 20 degrees (9-30 degrees) measured on post-operative radiographs (Figure 2 and Figure 3). Acceptable ranges of radiographic cup inclination angle were defined as abduction of 30-45 degrees and anteversion of 5-25 degrees [20,21]. Two cases (4%) had cup angles outside of the safe zones (Table 1 and Figure 4). There were no dislocations in these patients.

## Discussion

This case series assesses outcomes for the use of dual mobility bearings in revision total hip replacement. The main findings are as follows: (1) the rate of early dislocation was 1/55 (2%) cases; (2) there were no intra-prosthetic dislocations; (3) the rate of re-operation for any reason was 6/55 (11%) cases; (4) the post-operative infection rate was 2/55 (4%) cases; (5) medically related adverse events occurred in 2/55 (4%) cases; (6) the 90-day mortality rate was 1/55 (2%) cases; and (7) 2/55 (4%) cases had cup angles outside of the safe zones.

This case series demonstrates a low dislocation rate in the early post-operative period. This data is comparable to published literature on dual mobility bearings in revision total hip replacements [7,11]. A recent UK case series of revision total hip replacements using dual mobility bearings to treat recurrent instability demonstrated a re-dislocation rate of 0% in early- to mid-term follow-up [23]. Similarly, the rate of dislocation in revision total hip replacement surgery has been reported as low as 3.5% with dual mobility bearings [11]. A retrospective comparison of the dislocation rate following revision total hip replacement in obese patients found a dislocation rate of 15.6% with fixed implants compared with 0% with dual mobility bearings at one-year follow-up [24]. A meta-analysis of level 3 and 4 studies found a significantly lower odds of dislocation in revision surgery with dual mobility bearings compared to fixed implants [25]. Moreover, in a retrospective comparative study, dual mobility bearings had a significantly lower dislocation rate compared to large femoral heads in revision surgery [26]. A recent systematic review of studies involving dual mobility bearings in revision total hip replacement demonstrated an overall dislocation rate of 2.2% with dual mobility bearings versus 7.1% in the control group at a mean follow-up of 4.1 years [27].

Limitations of this study include its lack of long-term follow-up. Nonetheless, it has been reported that the majority of dislocations after revision total hip replacement occur within three months of surgery [28]. Polyethylene wear was a significant problem for first-generation dual mobility bearings, but newer-generation designs seem to have improved this issue over the short and medium term [12-15]. A recent systematic review of comparative studies concerning dual mobility bearings in primary and revision total hip replacement demonstrated excellent mid-term survivorship [27]. A meta-analysis of studies from five national joint registries demonstrated dual mobility construct all-cause survivorship of 97.8% at five years and 96.3% at 10 years [29]. It remains to be determined whether dual mobility bearings are low-wear implants in the long term.

This case series includes a range of indications for revision total hip replacement surgery. Revision surgery performed ranged from isolated acetabular component revision to total femoral replacement. Thus, the study results may be applicable to a broad population and may have good generalisability. This data is comparable to published literature regarding indications for revision total hip replacements. A retrospective study of 38,377 revision total hip replacements demonstrated that dislocation was the most common indication for revision surgery, followed by infection and aseptic loosening [30].

## Conclusions

This case series demonstrates a low dislocation rate in the early post-operative period for dual mobility bearings in revision total hip replacement. Based on current evidence, dual mobility bearings show promise as an early low dislocation implant in revision total hip replacement. However, further long-term follow-up data is needed to fully understand the effectiveness of dual mobility bearings in revision total hip replacement.
